# Imperceptible Flicker Noise Reduction Using Pseudo-Flicker Weight Functionalized Derivative Equalization in Light-Fidelity Transmission Link

**DOI:** 10.3390/s22228857

**Published:** 2022-11-16

**Authors:** Yong-Yuk Won, Jeungmo Kang

**Affiliations:** 1 Department of Electronic Engineering, Myongji University, 116 Myongji-ro, Cheoin-gu, Yongin 17058, Gyeonggi-do, Republic of Korea; 2 Product Technology Research Center, Korea Testing Certification Institute, Gunpo 15809, Gyeonggi-do, Republic of Korea

**Keywords:** derivative equalization, flicker noise, Li-Fi, pseudo-flicker weight function, white light emitting diode

## Abstract

A new technique to reduce flicker noise generated in light-fidelity (Li-Fi) transmission links based on the white light-emitting diode (LED) is proposed. Here, flicker noise with a frequency of 120 Hz, which is twice the frequency of AC power (60 Hz), is generated. The proposed technique is implemented in the receiver of the Li-Fi link. It can reduce flicker noise regardless of various digital modulation formats. In addition, there is no need to change the structure of the electrical circuit driving the LED to reduce the flicker noise. As a result, the non-return to-zero-on-off-keying (NRZ–OOK) signal waveform is tilted according to the flicker noise waveform. We implement the derivative equalization with a pseudo-flicker weight function to reduce the flicker noise. The derivative value of the NRZ–OOK signal mixed with flicker noise becomes larger than that without the flicker noise. In the proposed technique, the derivative value between adjacent sampling points is suppressed below the preset thresholds when it is greater than the preset threshold. Furthermore, a pseudo-flicker weight function is applied to accelerate the flicker noise reduction. As a result, using the proposed technique, a 2 dB signal-to-noise ratio (SNR) gain is obtained based on the bit error rate (BER) threshold (3.5 × 10^−5^) corresponding to 10% flicker modulation, which is known to have no serious effect on human health. This means that it is possible to implement a Li-Fi transmission link based on an illumination environment with a flicker modulation reduced from 10% to 7%.

## 1. Introduction

As the mobile communication network evolves from 5G to 6G, it is expected that the shortage of radio frequency (RF) resources will intensify [1,2]. Since various wireless communication services may be limited due to the lack of RF bandwidth, visible light communication (VLC) or light-fidelity (Li-Fi) technology can play a complementary role in the next-generation wireless transmission system in both the industrial and telecommunication field [3,4]. Furthermore, it has been recognized as one of the main alternatives [5,6]. In the Li-Fi system, after the white light emitting diode (LED) light is modulated by a digital or analog signal and then transmitted wirelessly, the modulated light is received by a photodetector.

Here, the flickering phenomenon of the LED may occur in the electric circuit driving the white LED and in the process of the white LED light being modulated by the signal [7,8]. Flicker is a phenomenon in which the brightness of light changes rapidly and repeatedly over time. The effects of flicker on human health range from reduced visual performance to nonspecific malaise to the development of some forms of epilepsy. The flicker can be divided into two main categories. It can be divided into visible flicker, which can cause epileptic seizures caused by direct light exposure for a few seconds, and imperceptible flicker, which can cause malaise, headache, and visual loss due to long-term indirect light exposure. Visible flicker generally corresponds to the flicker of visible light in the frequency range of from 3 Hz to 70 Hz, while imperceptible flicker refers to the invisible modulation of light at frequencies that the human eye cannot perceive [9,10]. This occurs in the form of a sinusoidal wave near 100 Hz or 120 Hz, which is twice the frequency of AC voltage [8,9,10]. It is easy to recognize the danger of visible flicker since it is immediately observed by the human eye. However, the risk of the imperceptible flicker may be higher because it is not well detected by the human eye. Since the transmission rate of the VLC or Li-Fi system that has been reported so far shows at least 10 Mb/s, the imperceptible flicker is more likely to occur than the visible flicker.

Existing techniques for reducing a flicker noise can be broadly classified into two categories [11,12,13,14,15,16,17]. One of them is to use various modulation formats that avoid the 120 Hz frequency where the flicker noise is located [11,12,13,14,15]. The other is a technique to design a circuit that drives an LED while suppressing the flicker noise [16,17]. Both techniques are implemented at the transmitter of the Li-Fi system. Techniques using the modulation format can reduce the transmission rate or complicate the hardware structure of the transmitter. The technique of implementing a driving circuit that reduces flicker noise can increase the cost of implementing a Li-Fi system, so it can slow down the commercialization of Li-Fi systems that need to implement lighting and communication functions at the same time. However, our proposed technique is located at the receiver of the Li-Fi system. It can reduce flicker noise regardless of various digital modulation formats. In addition, there is no need to change the structure of the electrical circuit driving the LED to reduce the flicker noise. To our knowledge, a technique to reduce flicker noise in the receiver is proposed for the first time. Furthermore, we know that a technique, which can reduce the flicker noise regardless of the modulation format of the received signal, is also proposed for the first time. Since the proposed technique is implemented based on digital signal processing (DSP) technology, a DSP-based circuit should be designed in the receiver when the Li-Fi link is realized in an actual network. Additionally, the waveform of the input signal should be a digital waveform such as on-off-keying (OOK) and pulse amplitude waveform (PAM) because the proposed technique only works well when the signal and noise have different waveforms. The above-mentioned descriptions are summarized as shown in Table 1. For your information, international standardization groups and European projects related to Li-Fi systems and flicker noise are as follows: Flicker mitigation solutions of PHYs in IEEE802.15.7 [18], Commercial Solutions for Classified (CSfC) for Li-Fi [19], and Enhance Lighting for the Internet of Things [20].

In this paper, we propose a technique to reduce the imperceptible flicker noises generated in the LED driving circuit and in the process of digital modulation such as non-return-to-zero-on-off-keying (NRZ–OOK) in a Li-Fi system based on white LED. In the Li-Fi transmission system, two types of imperceptible flicker occur at a frequency of 120 Hz. First, when the LED driver circuit is being operated, the flicker noise of 120 Hz, which is twice the AC voltage frequency, is generated. Second, when the NRZ–OOK signal is modulated, the flicker may occur as the baseband signal components, including the 120 Hz frequency, are modulated. Furthermore, the light from the white LED is directly modulated at the same time by the flicker noise generated in the LED driver circuit and the NRZ–OOK signal. The imperceptible flicker noise corresponding to a frequency of 120 Hz shows the waveform of a sinusoidal signal. We propose the following two techniques to reduce the imperceptible flicker noise. Firstly, since the change in the derivative value between adjacent sampling points of the flicker noise signal with an analog waveform is much larger than that of the NRZ–OOK signal having a digital waveform, the sampling points value corresponding to the derivative value over the preset threshold value are extracted to reduce the flicker noise signal. However, in the VLC transmission link, various optical noises other than flicker noise are also generated during wireless optical transmission. In addition, since these noises also represent a random analog waveform, various optical noises, and the flicker noise can be reduced together. In this case, since sampling points corresponding to various optical noises are extracted, the flicker noise reduction effect may be lowered. Therefore, a sinusoidal weight function with a frequency of 120 Hz and a random Gaussian phase is proposed additionally in order to maximize the reduction of the flicker noise. We named the proposed technique pseudo-flicker weight functionalized derivative equalization. In our previous work, a technique to reduce the interference noises and shot noises, which are produced in other illuminations existing around the Li-Fi link, was proposed and then the experimental results were presented [21]. My previous work was focused on reducing the interference noise and shot noise produced by the electric circuits that generate various lights (fluorescent lamps, halogen lamps, etc.). In the proposed technique, we focused on reducing the flicker noise generated in the wireless Li-Fi transmitter including the electric circuit that drives white LED. The important thing is that a pseudo-flicker weight function with the frequency of the flicker noise was proposed in order to increase the effect of reducing the flicker noise. A similarity to the previous work is as follows: It reduces the electrical noise generated in the electrical driver circuit. The differences and similarities between previous work and proposed techniques are summarized in Table 2. 

This paper is organized as follows. In Section 2, the proposed scheme for reducing flicker noise is described along with the mathematical equations. In Section 3, the implemented experimental Li-Fi link is presented to verify the proposed technique. In Section 4, the experimental results, which are measured using the implemented experimental Li-Fi link, are shown. Finally, Section 5 summarizes the content of the paper.

## 2. Pseudo-Flicker Weight Functionalized Derivative Equalization in Li-Fi Transmission

**Figure 1:**
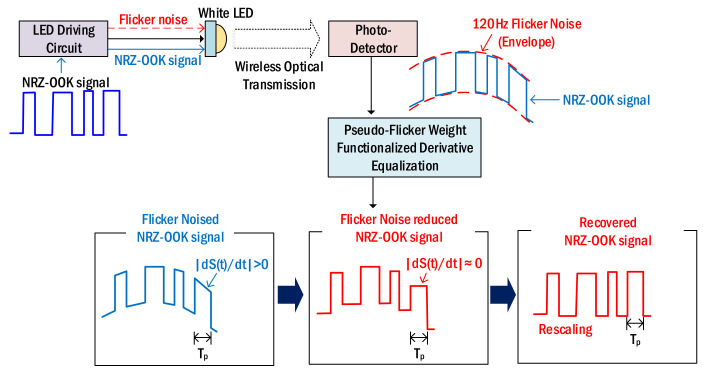
The 120 Hz flicker noise reduction using pseudo-flicker weight functionalized derivative equalization.

Figure 1 shows the concept of reducing 120 Hz flicker noise using the proposed technique. When the NRZ–OOK signal is injected into the LED driving circuit, the light from the white LED is simultaneously modulated by the flicker noise generated in the driving circuit and the NRZ–OOK signal. After the modulated LED light is transmitted wirelessly, the output signal, which is mixed 120 Hz flicker noise with NRZ–OOK signal, is produced at the photodetector. Here, since the frequency of the flicker noise is smaller than the maximum frequency of the NRZ–OOK signal, the flicker noise acts as an envelope and the NRZ–OOK signal is loaded on it. Therefore, as shown in the upper right of Figure 1, the intensity of the NRZ–OOK signal changes according to the change in the intensity of the flicker noise. In this case, the signal-to-noise ratio (SNR) of the NRZ–OOK signal may be reduced. We propose a pseudo-flicker weight functionalized derivative equalization technique to reduce the intensity of the flicker noise. The proposed technique reduces flicker noise through the following process. In the waveform of the NRZ–OOK signal mixed with flicker noise (flicker noised NRZ–OOK signal), the waveform of NRZ–OOK pulse corresponding to 0 (low) and 1 (high) is distorted according to the waveform of the flicker noise as shown in the bottom of Figure 1 (see the blue pulse). Here, if the waveform of the tilted NRZ–OOK pulse is recovered close to the input NRZ–OOK signal, the flicker noise can be reduced. At first, the absolute value of the derivative values between sampling points corresponding to the period of NRZ–OOK signal pulse (T_p_) is calculated to recover close to the input NRZ–OOK signal waveform. If the absolute difference between the calculated derivative values is greater than the preset threshold, the intensity of the corresponding sampling point is suppressed. This process is repeated until the absolute difference between derivative values becomes smaller than the preset threshold.

The flicker noise reduction can be summarized as follows using mathematical equations. The NRZ–OOK symbol can be described as in Equation (1).

(1) $$s\left(i\right)=\sum_{k=1}^{N}B\left(k,i\right)g\left(\frac{\left(t\left(i\right)-kT\right)}{T}\right),g\left(\alpha \right)=\left\{\begin{array}{c} 1,\;\;\;\;for\;0\leq \alpha <1\;\;\\ 0,\;\;\;\;\;\;\;\;\;\;\;\;otherwise\; \end{array}\right.$$

where $B\left(k,i\right)$ is a pseudo-random bit sequence (PRBS), $g\left(\alpha \right)$ is an NRZ–OOK pulse. N is the total number of NRZ–OOK symbols. The flicker noise corresponding to the NRZ–OOK symbol can be expressed in the form of a sinusoidal function as in Equation (2).

(2) $$e\left(i\right)=C\left(i\right)exp\left(j2\pi f_{0}t\left(i\right)+\phi \left(i\right)\right)$$

where $C\left(i\right)$ is an intensity of flicker noise at the sampling point of i. M is the total number of sampling points for one symbol. N is the total number of NRZ–OOK symbols. $f_{0}$ is the frequency of flicker noise. $\phi \left(i\right)$ is a random phase uncorrelated for each i, which is generated due to the use of the LED driving circuit.

Next, the light from white LED, which is directly modulated by NRZ–OOK symbol and flicker noise, can be written as Equation (3).

(3) $$A_{s}\left(i\right)=\left(s\left(i\right)+e\left(i\right)\right)\sum_{m=-\prod_{0}}^{\prod_{0}}exp\left\{j(2\pi \left(\nu_{0}+m\delta \nu_{0}\right)t\left(i\right)+\Phi \left(m\right)\right\}$$

where $\nu_{0}$ is the center optical frequency in the spectral region of white LED light. $\delta \nu_{0}$ is the elemental frequency spacing of the white LED light. Φ is random optical phase uncorrelated for each m. When $B_{0}$ is the total spectral width of white LED light, $\prod_{0}$ is $\frac{B_{0}}{2\delta \nu_{0}}$.

When the modulated light is received at the photodetector, four different kinds of signals (NRZ–OOK signal, flicker noise, and two different shot noises) are generated. One is a non-varying stationary shot noise generated by the photodetector itself, and the other is a time-varying and non-stationary shot noise generated due to the direct modulation of the NRZ–OOK signal. Therefore, the output photocurrent after the photodetector can be expressed as Equation (4).

(4) $$\begin{array}{cc} I_{r}\left(i\right) & =R\sigma_{L}{\left\{\frac{A_{s}\left(i\right)}{\sqrt{2}}\right\}}^{2}\\ & =\frac{R}{2}\sigma_{L}\overset{N}{\underset{k=1}{\sum }}B\left(k,i\right)g\left(\frac{\left(t\left(i\right)-kT\right)}{T}\right)+\frac{R}{2}\sigma_{L}C\left(i\right)exp\left(j2\pi f_{0}t\left(i\right)+\phi \left(i\right)\right)\\ & \;+\frac{R^{2}\sigma_{L}\underset{M=\infty }{\lim}\sum_{n=1}^{M}A_{s}{}^{2}\left(n\right)h_{I}^{2}\left(t\left(i\right)-\tau \left(n\right)\right)}{8B}+\frac{R}{2}\sigma_{L}I_{shot} \end{array}$$

where R is the responsivity of the photodetector. $\sigma_{L}$ is the optical loss during the optical wireless transmission. $h_{I}$ is the impulse response of the photodetector with transimpedance amplifier. B is 3-dB bandwidth of photodetector. $I_{shot}$ is a non-varying stationary shot noise, which is produced by photodetector. The first term on the right side of Equation (4) is an NRZ–OOK signal having a digital waveform, and the second term is a flicker noise signal having an analog waveform. The third term on the right side of Equation (4) means the time-varying and non-stationary shot noise current, which is generated by the direct modulation of NRZ–OOK signals. This is implemented using Personick analysis [22]. It is well known that this non-stationary shot noise has a white spectral characteristic that correlates with the modulated signal and shows a Poisson probability distribution [23]. Here, since the frequency of the imperceptible flicker noise, $f_{0}$ (around 120 Hz), is usually within the NRZ–OOK signal spectrum, it is very difficult to reduce the flicker noise using a software-based finite impulse response (FIR) filter or a hardware-based matched RF filter. Therefore, we propose a pseudo-flicker weight functionalized derivative equalization technique to reduce the imperceptible flicker noise. The proposed technique is implemented based on the conventional total variation (TV) technique. However, since the conventional TV technique focuses on reducing random analog noise having a Gaussian distribution function as a variance, it may not be able to reduce the flicker noise with a sinusoidal waveform effectively. Accordingly, we implemented a non-local weight function with a random Gaussian phase exhibiting characteristics similar to the flicker noise and then adapted it to the conventional TV technique in order to maximize the flicker noise reduction. In this way, the proposed technique focuses more on reducing flicker noise than on random analog noise such as shot noise. The proposed technique can be expressed in Equation (5) as follows.

(5) $$z_{p}\left(i\right)=\underset{f_{p}\in H_{f_{p}}}{\mathrm{argmin}}\sum_{\tau \in \mathbb{R}\left(i\right)}{\left|f_{p}\left(i\right)-\tilde{S\left(\tau \right)}\right|}^{2}\Theta \left(\tau ,i\right)+\lambda {∥f_{p}∥}_{TV}$$

where $z_{p}\left(i\right)$ is a polynomial function of degree p or less. $\tilde{S\left(\tau \right)}$ is a received NRZ–OOK signal adding the flicker noise. $H_{f_{p}}$ is the full set of all polynomial functions of degree p or less. $f_{p}$ is one of the polynomial functions in the full set, $H_{f_{p}}$. λ is a regularization parameter that controls the waveform of the recovered NRZ–OOK signal. ${∥\;\;∥}_{TV}$ is TV norm, ${∥f_{p}∥}_{TV}=\int_{\mathbb{R}}^{}\left|\nabla f_{p}\right|$ with the gradient operator, ∇. $\Theta \left(\tau ,i\right)$ is a weight function with a random Gaussian phase that exhibits very similar properties to imperceptible flicker noise as shown in Equation (6).

(6) $$\Theta \left(\tau ,i\right)=exp\left\{-j2\pi f_{0}\left(\frac{\sum_{\delta \in \Gamma }∥I_{r}\left(\tau +\delta \right)-I_{r}{\left(i+\delta \right)∥}^{2}G_{\sigma }(∥\delta ∥)}{h^{2}}\right)\right\}$$

where $G_{\sigma }$ is the Gaussian distribution function with a standard deviation of σ. Γ is a subset of the sampling points located around sampling points of i and τ. h is a filtering parameter. As shown in Equation (6), a weight function, which is changed with the period corresponding to the flicker noise frequency, is implemented by multiplying the Gaussian distribution function and the differential values between the adjacent sampling points of the received photocurrent in order to further reduce the intensity of the sampling points corresponding to the flicker noise. The mathematical process of Equation (5) for obtaining the NRZ–OOK signal with reduced flicker noise is as follows. The derivative values between an arbitrary polynomial function, $f_{p}\left(i\right)$ and the received signal, $\tilde{s\left(\tau \right)}$ are multiplied by the weight function, $\Theta \left(\tau ,i\right)$. After that, the iterative operations of the derivative are executed until the multiplied values become smaller than the preset threshold. In this process, except for the digital waveform, the intensity of the sampling points corresponding to the analog waveform becomes smaller and smaller. Here, the intensity of sampling points corresponding to the flicker noise signal is further reduced due to the use of the weight function exhibiting the characteristics of the flicker noise. In addition, the value multiplied by the regularization parameter, λ and the norm of the polynomial function, $f_{p}\left(i\right)$ is added in order to control the waveform of the polynomial function, $z_{p}\left(i\right)$. Finally, the polynomial function, $z_{p}\left(i\right)$ obtained in Equation (5) corresponds to the recovered NRZ–OOK signal with reduced flicker noise.

## 3. Experimental Setup

**Figure 2:**
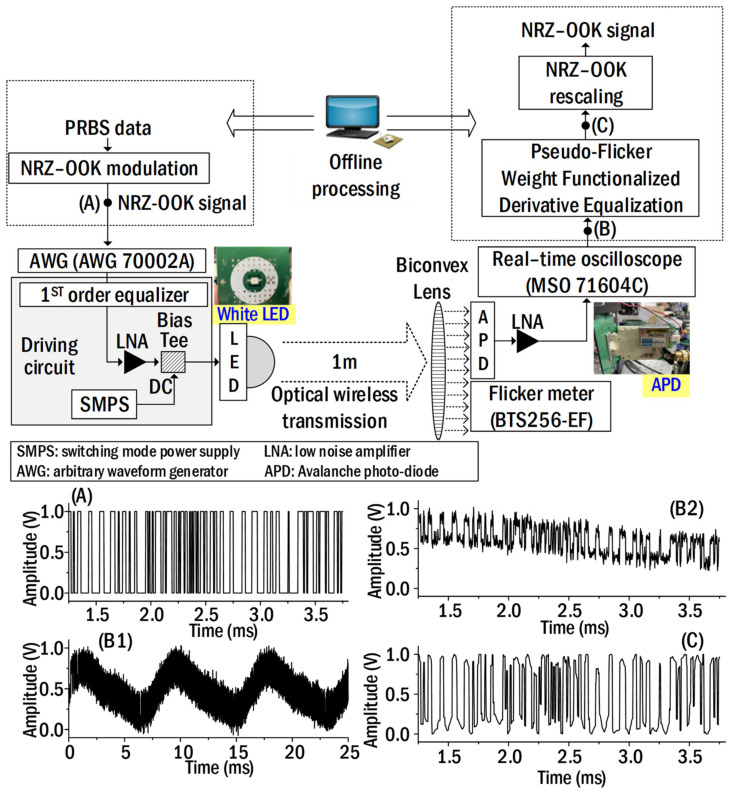
Experimental setup: Li-Fi link for the reduction of flicker noise. (**A**) Input NRZ-OOK waveform, (**B1**) Overall waveform of received signal, (**B2**) Received signal waveform of 250bits (**C**) Recovered signal waveform after flicker noise reduction.

Figure 2 shows the experimental Li-Fi link, which was implemented to experimentally verify the proposed technique for reducing flicker noise. In the implemented Li-Fi link, the maximum light output after 1 m optical wireless transmission was 900 Lux. The frequency response of the Li-Fi link was about 10 MHz. Both NRZ–OOK signal generation and flicker noise reduction in the area corresponding to the dashed box are handled offline using MATLAB. The NRZ–OOK signals are produced using pseudo-random bit sequence (PRBS) with length of 2^17^−1. The number of samples per bit was set to 4. The bandwidth of NRZ–OOK signal was 100 kHz. The NRZ–OOK signal was loaded onto an arbitrary waveform generator (AWG: AWG70002A, Tektronix) sampled at 400 kS/s. The NRZ–OOK signal from the AWG was amplified using the low noise amplifier (LNA: PE15A63012, Pasternack). The light from the white LED (LUW-W5AM, OSRAM^®^, Munich, Germany) was directly modulated by the NRZ–OOK signal biased using a bias-tee. The used white LED was manufactured in surface mount technology (SMT) package type. Its forward voltage was 3.2 V. It was implemented based on ThinGaN. Its light output was 116 lm and the viewing angle of 50% light output was 170°. The switch-mode power supply (SMPS), which was used to apply DC voltage to white LED, employed 220 V AC power as input power and then selectively generates output DC voltage from 3 V to 12 V. The modulated light was transmitted wirelessly over a distance of 1 m and then received at the Avalanche Photodetector (APD: C5331-11, Hamamatsu, Shizuoka, Japan, 100-MHz bandwidth) after a biconvex lens (LB1723-A, Thorlabs, Jessup, MD, USA). Since the light generated from the white LED has a diffuse property, the amount of light received at the photodetector decreases as the transmission distance increases. Therefore, it is necessary to increase the amount of received light. We used a biconvex lens to increase the amount of the received light. The role of the biconvex lens is to collect the diffused light so that the amount of light received at the photodetector is maximized. The specification of the biconvex lens is as follows. Its shape was biconvex type, and the focal length was 60 mm. Its diameter was 50.8 mm. The wavelength passband of light was 350 to 700 nm. The used APD operates at 5 V, and the wavelength range of the received optical signal was from 400 nm to 1000 nm. The maximum optical power that the APD could receive without saturation was 2000 Lux. In addition, the flicker modulation of the transmitted light was measured using a flicker meter (BTS256-EF, Gigahertz-Optik, Türkenfeld, Germany). Here, the flicker modulation is an index indicating the intensity of the flicker noise [8]. The flicker modulation is obtained using Equation (7) by measuring the maximum luminance and minimum luminance values during one period of flicker noise in the light emitted from the white LED [8].

(7) $$F_{m}=100\times \frac{L_{max}-L_{min}}{L_{max}+L_{min}}$$

where $F_{m}$ is a flicker modulation in units of percentage. $L_{max}$ is a maximum luminance, and $L_{min}$ is a minimum luminance within one period. The waveform of the received signal was captured using a real-time oscilloscope (MSO 71604C, Tektronix, Beaverton, OR, USA) sampled at 1.6 MS/s and then stored on a computer. The regularization parameter was 5. We recovered the NRZ–OOK signal from the stored waveform using the proposed technique. Next, the recovered NRZ–OOK signal waveform has been finely adjusted according to the input signal level. For your information, the proposed technique was implemented by offline processing in the dashed box. The waveforms at the bottom of Figure 2 show the NRZ–OOK signal waveforms measured at each point. As shown in the waveform measured at point (A), the input NRZ–OOK signal waveform was generated correctly. In the waveform measured from the real-time oscilloscope (point (B)), we found that the NRZ–OOK signal waveform was loaded on the flicker noise. The flicker noise was generated by AC-powered SMPS. Additionally, we presented the waveform corresponding to 250 bits of the total length of the NRZ–OOK signal on the right in order to clearly show the effect of the flicker noise and the shape of the restored waveform using the proposed technique. As shown in the 250–bits waveform, it was found that the waveform was tilted by the flicker noise (upper figure in the right waveform). However, as shown in the lower waveform (point (C)), both high-frequency noise and flicker noise were reduced using the proposed technique.

## 4. Experimental Results

**Figure 3:**
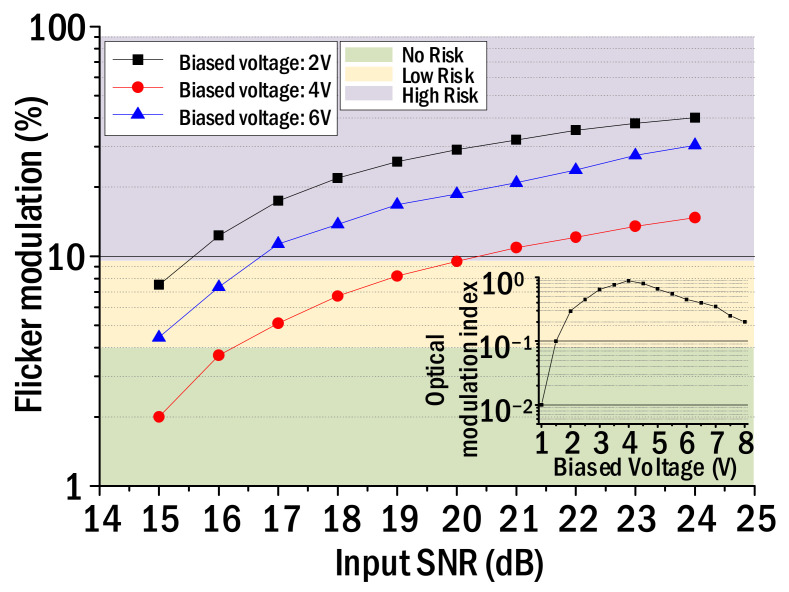
Flicker modulation against the SNR change in input NRZ–OOK signal.

Figure 3 shows the flicker modulation, which was measured by changing the SNR of the input NRZ–OOK pulse at each biased voltage. The flicker modulation was measured repeatedly using the flicker meter. The inset in Figure 3 shows the measured optical modulation index (OMI) of white LED while changing the bias voltage. The OMI is defined as the ratio of half the peak-to-peak optical signal power and the average optical power. We calculated the OMI by measuring the intensity of the sinusoidal signal just before its output signal waveform is clipped in the process of modulating the light of the white LED by the sinusoidal signal. As shown in the inset of Figure 3, the maximum optical modulation index was observed when the biased voltage was around 4 V. According to IEEE Standard PAR 1789, there is no risk to health if the flicker modulation does not exceed 4% or less at 120 Hz flicker frequency, and the flicker modulation from 4% to 10% indicates low risk [7]. Importantly, if the Flicker Modulation Index exceeds 10%, it means that a person may be exposed to high health risks [7]. Therefore, IEEE Standard 1789 recommends that the light of the white LED be modulated under the operating condition showing a flicker modulation index of 10% or less. As shown in Figure 3, it can be seen that the input SNR can be set up to about 20 dB when the biased voltage is 4 V based on the 10% flicker modulation. In addition, the result of Figure 3 tells us that it is possible to minimize the flicker effect on health when the input SNR is set up to 15.5 dB and 16.5 dB, respectively, at the bias voltage of 2 V and 6 V. This means it is possible to transmit an NRZ–OOK signal with about 4.5 dB greater near the 4 V biased voltage than when the biased voltage is 2 V. The reason why the light from the white LED can be modulated directly by the NRZ–OOK signal with the highest SNR at the biased voltage (4 V) corresponding to the maximum OMI is as follows. If the NRZ–OOK signal is optically modulated at a biased voltage with a low optical modulation index, the output signal waveform can be clipped and distorted as the SNR of the input signal increases. Due to the optical distortion, not only does the intensity of 120 Hz flicker noise increase but also harmonic components of the flicker noise are also generated. Therefore, it is found that the flicker modulation is inversely proportional to the OMI. Therefore, it is important to minimize the intensity of flicker noise by modulating the NRZ–OOK signal in the section where the optical modulation index is maximized.

**Figure 4:**
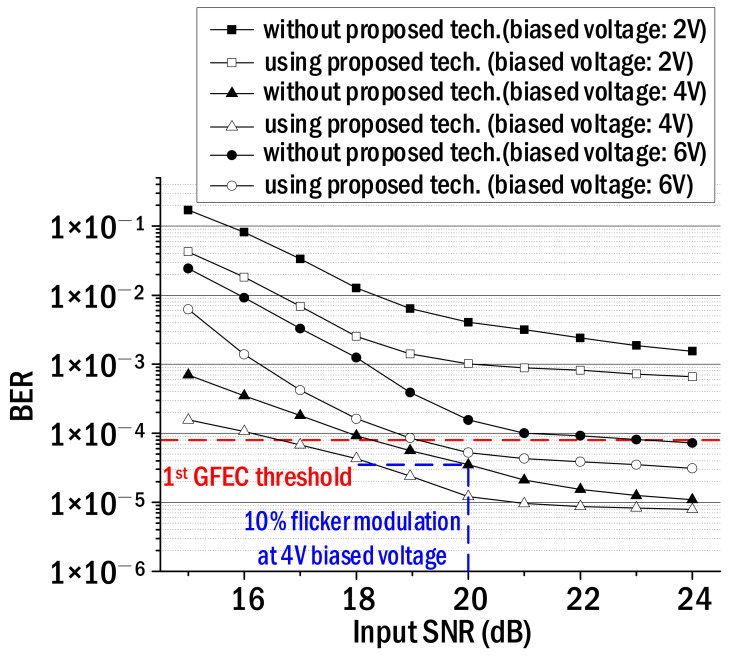
BER of NRZ–OOK signal vs. input SNR.

Figure 4 shows the bit error rates (BERs) before and after employing the proposed technique, which were measured repeatedly while increasing the SNR of the input NRZ–OOK signal from 15 dB to 24 dB. In the legend of Figure 4, the filled shapes represent the BERs in the case of not using the proposed technique, and the open shapes correspond to the BERs when the proposed technique is applied. The filled squares and the empty squares are the measured BERs when the bias voltage is 2 V. The filled triangles and the empty ones are the BERs when the bias voltage is 4 V while the filled and empty circles represent the measured BER when the bias voltage is 6 V. As shown in Figure 4, the BER decreased as the SNR of the input signal increased, and then the BER floor was observed as the SNR exceeded 21 dB. The reason for the BER floor is that even if the SNR increases, the SNR of the output NRZ–OOK signal does not increase continuously and is saturated due to the OMI determined according to the biased voltage. Furthermore, when the bias voltage is 4 V, the BER floor was observed even if the SNR of the input NRZ–OOK signal went up because BERs smaller than the minimum BER (7.63 × 10^−6^) corresponding to the PRBS length could not be obtained. Another important point is that when the bias voltage is 4V, about 1.5-dB SNR gain can be obtained using the proposed technique based on the 1st general forward error correction (GFEC) BER threshold. In other words, it is possible to transmit the NRZ–OOK signal if its SNR is 17 dB in the case of using the proposed technique. However, the SNR of 18.5 dB or more is required to transmit the NRZ–OOK signal when the proposed technique is not utilized. Additionally, as mentioned in Figure 3, the SNR corresponding to 10% flicker modulation at 4 V biased voltage was 20 dB. In this case, the BER without using the proposed method was 3.5 × 10^−5^ as shown in Figure 4. Therefore, the BER (3.5 × 10^−5^) corresponding to 10% flicker modulation is obtained using the proposed technique even when the SNR of the input NRZ–OOK signal is set to 18 dB, not 20 dB. This result tells us that optical wireless transmission is possible even in an illuminance environment where the flicker modulation is reduced from 10% to 7%.

**Figure 5:**
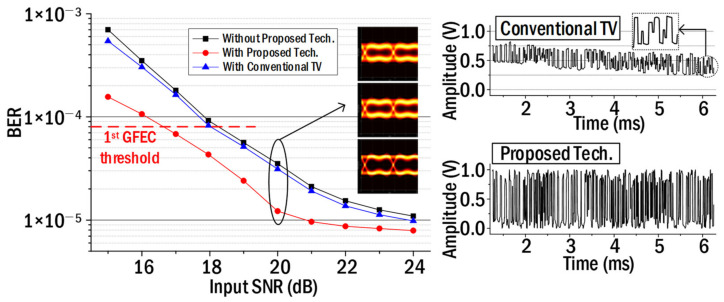
BER comparison between the proposed technique and conventional TV.

It is necessary to verify the effect of the pseudo-flicker weight function mentioned in Equation (6) in order to remove the flicker noise. So, we compared the performance between the conventional total variation (TV) technique and the proposed technique. The technique proposed by Rudin was employed as the conventional TV [24]. Figure 5 shows the measured BERs for the proposed technique and the conventional TV, respectively. The inserted eye patterns in Figure 5 show the one in the case of not using the proposed technique, the one in the case of using the conventional TV, and the one in the case of using the proposed technique from the top in order. The signal waveform located on the right side of Figure 5 shows the NRZ–OOK signal waveforms in the case of using the general TV technique and the proposed technique. In the case of using the general TV, the high-frequency noises were reduced (refer to the enlarged waveform near 6 ms). However, it was found that the 120 Hz flicker noise was maintained because the overall signal waveform was tilted. In the case of using the proposed technique, it could be seen that the NRZ–OOK signal waveform was uniformly recovered without inclination due to the pseudo-flicker weight function. In addition, as shown in Figure 5, an SNR gain of about 1.5 dB was obtained using the proposed technique compared to the conventional TV at the 1st GFEC BER threshold.

**Figure 6:**
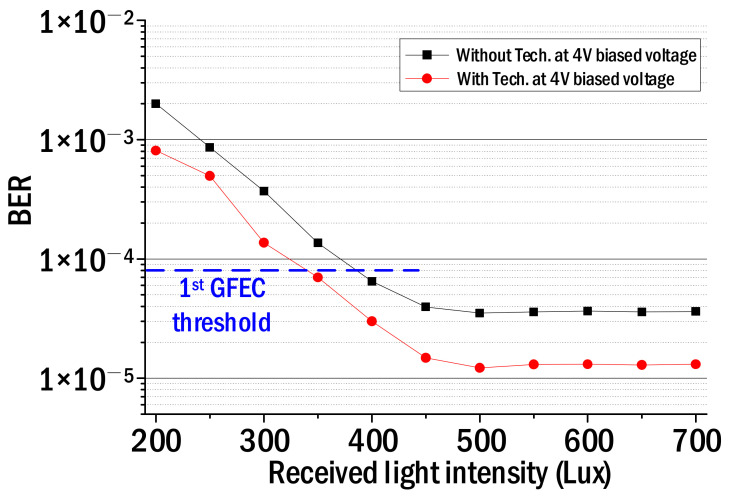
BER of NRZ–OOK signal vs. received light intensity.

Figure 6 shows the measured BERs while increasing the received optical power from 200 Lux to 700 Lux. At this time, the biased voltage was set to 4 V and the input SNR was set to 20 dB. Filled squares show the BER when the proposed technique is not applied, and filled red circles show the BER when the proposed technique is applied. As shown in Figure 6, it was found that the BER corresponding to the first-generation GFEC threshold can be obtained even if the received light intensity is reduced by 50 Lux using the proposed technique. Reducing the received light intensity means less likely to create an illuminating environment with flicker noise. The BER floor was also observed when the received light intensity exceeded 500 Lux. This is because the signal-to-noise ratio of the recovered NRZ–OOK signal does not increase any more even when the received light intensity increases.

## 5. Conclusions

We proposed a differential equalization technique with a pseudo-flicker weighting function to reduce the imperceptible flicker noise generated in the Li-Fi transmission link. The imperceptible flicker noise was generated in the white LED driving circuit and mainly has a frequency of 120 Hz (in the case of 220–V AC power). It was modulated simultaneously with the NRZ–OOK signal in the process of directly modulating the light of the white LED. As a result, the 120 Hz imperceptible flicker noise plays the role of reducing the SNR of the NRZ–OOK signal. Using the proposed technique, the following main experimental results were obtained. The communication and illumination environment of the implemented optical wireless link is as follows. At a bias voltage of 4 V with maximum OMI, the light of the white LED was directly modulated by the NRZ–OOK signal with an SNR of 20 dB (corresponding to 10% flicker modulation). After that, the modulated light was transmitted wirelessly by 50 cm and then the received light intensity was 500 Lux. A 2 dB SNR gain was obtained using the proposed technique based on the BER threshold (3.5 × 10^−5^) corresponding to 10% flicker modulation, which is known to have no significant impact on human health. This means that even if the input SNR is set to 18 dB from 20 dB, the BER corresponding to 10% flicker modulation can be obtained using the proposed technique as shown in Figure 4.

Furthermore, it means that it is possible to implement an illumination and communication environment with flicker modulation reduced from 10% to 7%. Therefore, it can be said that it is possible to realize an illuminating Li-Fi space with attenuated flicker noise using the proposed technique. In addition, the proposed technique is expected to be applicable to other fields. For example, speckle noises are known to degrade performance in laser projection systems, and many techniques have been proposed to reduce them [25,26]. In terms of reducing analog signals mixed with digital signals, the proposed technique can be applied to reduce speckle noises generated in laser projection systems. As in the example mentioned above, it is necessary to analyze whether the proposed technique in other fields can be employed in terms of scalability. Considering the proposed technique in terms of human life, it is possible to build a Li-Fi network in which the flicker effect does not affect human health because the proposed technique can reduce the flicker noise generated in the Li-Fi system.

## Figures and Tables

**Table 1:** Comparison between the existing techniques and the proposed technique.

Technique	Advantages and Weakness	Location
Modulation format to avoid flicker noise	Flicker noise elimination Transmission rate reduction High hardware complexity A circuit or algorithm that recovers a specific modulated formatted signal	Transmitter in Li-Fi system
LED driver circuit for flicker noise reduction	Flicker noise reduction High implementation cost	Transmitter in Li-Fi system
Proposed technique	Flicker noise reduction regardless of digital modulation format DSP (digital signal processing) implementation Signals and noise with different waveforms	Receiver in Li-Fi system

**Table 2:** Comparison between previous work and the proposed technique.

Technique	Differences	Similarities
Previous work [21]	Reduction of the interference noise and shot noise produced by the electric circuits that generate various lights around Li-Fi link	Reduction of the electrical noises generated in the electrical driver circuit
Proposed technique	Reduction of the flicker noise generated in the wireless Li-Fi transmitter including the electric circuit that drives white LED	

## Data Availability

Not applicable.

## References

[1] Hass H. (2017). LiFi is a paradigm-shifting 5G technology. Rev. Phys..

[2] Albraheem L.I., Alhudaithy L.H., Aljaser A.A., Aldhaan M.R., Bahliwah G.M. (2018). Toward designing a Li–Fi-based hierarchical IoT architecture. IEEE Access.

[3] Arai S., Kinoshita M., Yamazato T. (2021). Optical wireless communication: A candidate 6G technology?. IEICE Trans. Fundam. Electron. Commun. Comput. Sci..

[4] Ariyanti S., Suryanegara M. Visible light communication (VLC) for 6G technology: The potency and research challenges. Proceedings of the 2020 Fourth World Conference on Smart Trends in Systems, Security and Sustainability (WorldS4).

[5] Wu X., Soltani M.D., Zhou L., Safari M., Haas H. (2021). Hybrid LiFi and WiFi Networks: A Survey. IEEE Commun. Surv. Tutor..

[6] Chowdhury M.Z., Hasan M.K., Shahjalal M., Hossan M.T., Jang Y.M. (2020). Optical Wireless Hybrid Networks: Trends, Opportunities, Challenges, and Research Directions. IEEE Commun. Surv. Tutor..

[7] Wilkins A., Veitch J., Lehman B. LED lighting flicker and potential health concerns: IEEE standard PAR1789 update. Proceedings of the 2010 IEEE Energy Conversion Congress and Exposition.

[8] (2015). IEEE Recommended Practices for Modulating Current in High-Brightness LEDs for Mitigating Health Risks to Viewers.

[9] Burns S.A., Elsner A.E., Kreitz M.R. (1992). Analysis of nonlinearities in the flicker ERG. Optom. Vis. Sci..

[10] Cai R., Cobben J.F.G., Myrzik J.M.A., Blom J.H., Kling W.L. (2009). Flicker response of different lamp types. IET Gener. Transm. Distrib..

[11] Mejia C.E., Georghiades C.N., Abdallah M.M., Al-Badarneh Y.H. (2017). Code design for flicker mitigation in visible light communications using nite state machines. IEEE Trans. Commun..

[12] Oh M. A flicker mitigation modulation scheme for visible light communications. Proceedings of the 2013 15th International Conference on Advanced Communications Technology (ICACT).

[13] Fang J., Che Z., Jiang Z.L., Yu X., Yiu S.-M., Ren K., Tan X., Chen Z. (2017). An efficient flicker-free FEC coding scheme for dimmable visible light communication based on polar codes. IEEE Photonics J..

[14] Han Y., Kim Y., Kim B.W. (2019). Bit-Shuffle Coding for Flicker Mitigation in Visible Light Communication. IEEE Access.

[15] Liu L., Chen L.K. Four-level flicker-mitigation coding scheme in the non line-of-sight optical camera communication system. Proceedings of the Optoelectronics and Communications Conference (OECC).

[16] Sun L., Han Y. (2021). A capacitorless and low-optical-flicker AC direct LED driving IC and system applied to street lighting. IEEJ Trans. Electr. Electron. Eng..

[17] Chong K.H., Gao Y., Mok P.K.T. (2021). A customized AC hybrid LED driver with flicker reduction for high nominal range applications. IEEE Trans. Circuits Syst. II Express Briefs.

[18] IEEE P802.15 Working Group for Wireless Personal Area Networks (WPANs): Flicker Mitigation solutions of PHYs in IEEE802.15.7. https://www.ieee802.org/15/pub/TG13.html.

[19] IEEE P802.11 Light Communications Study Group: Commercial Solutions for Classified (CSfC) for Li-Fi. https://www.ieee802.org/11/.

[20] ELIoT: Enhance Lighting for the Internet of Things. www.eliot-h2020.eu.

[21] Won Y.-Y., Yoon S.M., Seo D. (2021). Ambient LED Light Noise Reduction Using Adaptive Differential Equalization in Li-Fi Wireless Link. Sensors.

[22] Personick S.D. (1973). Receiver design for digital fiber optic communication systems, II. Bell Syst. Tech. J..

[23] Pang G., Zhou Y. (2018). Functional limit theorems for a new class of non-stationary shot noise processes. Stoch. Process. Appl..

[24] Rudin L.I., Osher S., Fatemi E. (1992). Nonlinear total variation based noise removal algorithms. Phys. D.

[25] Yılmazlar I., Sabuncu M. (2015). Speckle noise reduction based on induced mode Hopping in a semiconductor laser diode by drive current modulation. Opt. Laser Technol..

[26] Yılmazlar I., Sabuncu M. (2015). Implementation of a Current Drive Modulator for Effective Speckle Suppression in a Laser Projection System. IEEE Photonics J..

